# A novel flow waveform partitioning method based on the HeartCon and its clinical application

**DOI:** 10.3389/fcvm.2025.1649269

**Published:** 2026-03-24

**Authors:** Haosong Wang, Gang Lin, Zhifu Han, Xiangyu Liu

**Affiliations:** Department of Clinical Research, Aerospace Taixin Technology Co., Tianjin, China

**Keywords:** animal experiment, cardiac cycle, diastolic phase, ejection fraction, flow waveform, HeartCon, left ventricular assist device, Wiggers diagram

## Abstract

Left ventricular assist device (LVAD) is a crucial therapy for end-stage heart failure. This research aimed to validate the correspondence between the HeartCon flow waveform and the cardiac cycle through animal experiments and propose a novel electrocardiogram (ECG)-guided partitioning method. The controlled variable method was employed to systematically investigate the effects of varying rotational speeds, heart rates, and blood pressures on the HeartCon flow waveform and the cardiac cycle. The results demonstrated that the proposed HeartCon partitioning method exhibited higher accuracy in assessing ejection fraction (EF), with absolute relative errors below 5% when compared to echocardiography, significantly outperforming the HeartWare Ventricular Assist Device (HVAD)-based partitioning method (absolute relative errors exceeding 40%). Furthermore, the study revealed that the diastolic phase proportion was consistently less than 58% of that in a normal heart under various physiological conditions and further shortened with increased heart rate, elevated rotational speed, and decreased blood pressure, suggesting a potential significant impact of aspiration force produced by the pump on the diastolic phase. Based on the experimental findings, a HeartCon cardiac Wiggers diagram was constructed, clearly illustrating the temporal relationship and key characteristics of pump flow, left ventricular pressure, and aortic pressure after LVAD implantation. This novel flow waveform partitioning method provides a theoretical foundation for the clinical application of the HeartCon pump and offers new avenues for clinicians to monitor and assess patients’ physiological conditions through real-time flow waveforms. AI Assistance Declaration: During the preparation of this work, the authors used ChatGPT (OpenAI) to improve language and readability. After using this tool, the authors reviewed and edited the content as needed and take full responsibility for the publication's content.

## 1 Introduction

Heart failure (HF) poses a significant threat to patients' quality of life. According to the China Cardiovascular Health and Disease Report, in 2022, 5,402 hospitals across China admitted a total of 10.29 million heart failure (HF) patients. The mean age of hospitalized HF patients was 71.0 ± 12.7 years. The in-hospital mortality rate for heart failure patients was 2.6%, while the non-rehabilitative discharge rate was 10.2%, and the 30-day readmission rate was 10.0% (1). The American Heart Association reports that in 2020, the prevalence of heart failure in adults in the United States was approximately 1.9%–2.6%, with about 6.7 million Americans aged 20 years or older living with HF, and this number is projected to rise to 2.97%, affecting about 8.5 million Americans by 2030 (2). Left ventricular assist devices (LVADs) are a critical treatment modality for end-stage HF, providing mechanical circulatory support by unloading the left ventricle and maintaining cardiac output. LVAD therapy has evolved significantly, with continuous-flow pumps now representing the standard of care due to their improved durability and smaller size compared to earlier pulsatile devices (3). These devices not only serve as a bridge to transplantation but are also increasingly used as destination therapy, significantly improving survival and functional status in carefully selected patients (4). The fundamental principle involves diverting blood from the left ventricle to the ascending aorta via an inflow cannula and an outflow graft, thereby reducing ventricular workload and improving end-organ perfusion (5). Continuous-flow LVADs, such as the HeartWare Ventricular Assist Device (HVAD) and HeartCon, have improved survival and quality of life in advanced heart failure patients. Among the LVADs approved in the United States, the HVAD uniquely offered the capability of providing instantaneous calculated flow waveforms, the analysis of which could yield information about the patient's physiological status and the pump's operational state (6). However, the discontinuation of the HVAD in 2021 due to safety concerns including a higher incidence of neurological adverse events and pump thrombosis (7) has limited ongoing research and the accumulation of clinical experience based on its flow waveforms, highlighting the importance of developing a leading LVAD capable of computing and displaying flow waveforms, as well as novel methodologies for flow waveform analysis.

Rocor Medical has independently developed the HeartCon pump, which is fully protected by proprietary intellectual property rights. It incorporates an innovative design in magnetic fluid suspension technology, achieving enhanced reliability and biocompatibility. Its integrated pump-motor design also simplifies the implantation procedure. Various *in vitro* experimental indicators have reached internationally comparable levels, and it successfully obtained a registration certificate from the National Medical Products Administration of China in July 2022. Clinical staff can understand the patient's current physiological condition by observing the real-time pump flow waveform displayed on the HeartCon system's monitor. While a pressure-volume (PV) loop analysis could provide deeper insights into the differential physiological effects of various LVADs (including HeartCon and HVAD) on ventricular unloading and energetics, such an analysis is beyond the scope of this manuscript, which focuses primarily on establishing and validating a novel flow waveform partitioning method. Future studies incorporating PV loop measurements are planned to further elucidate the comparative physiology of different LVAD systems.

**Figure 1:**
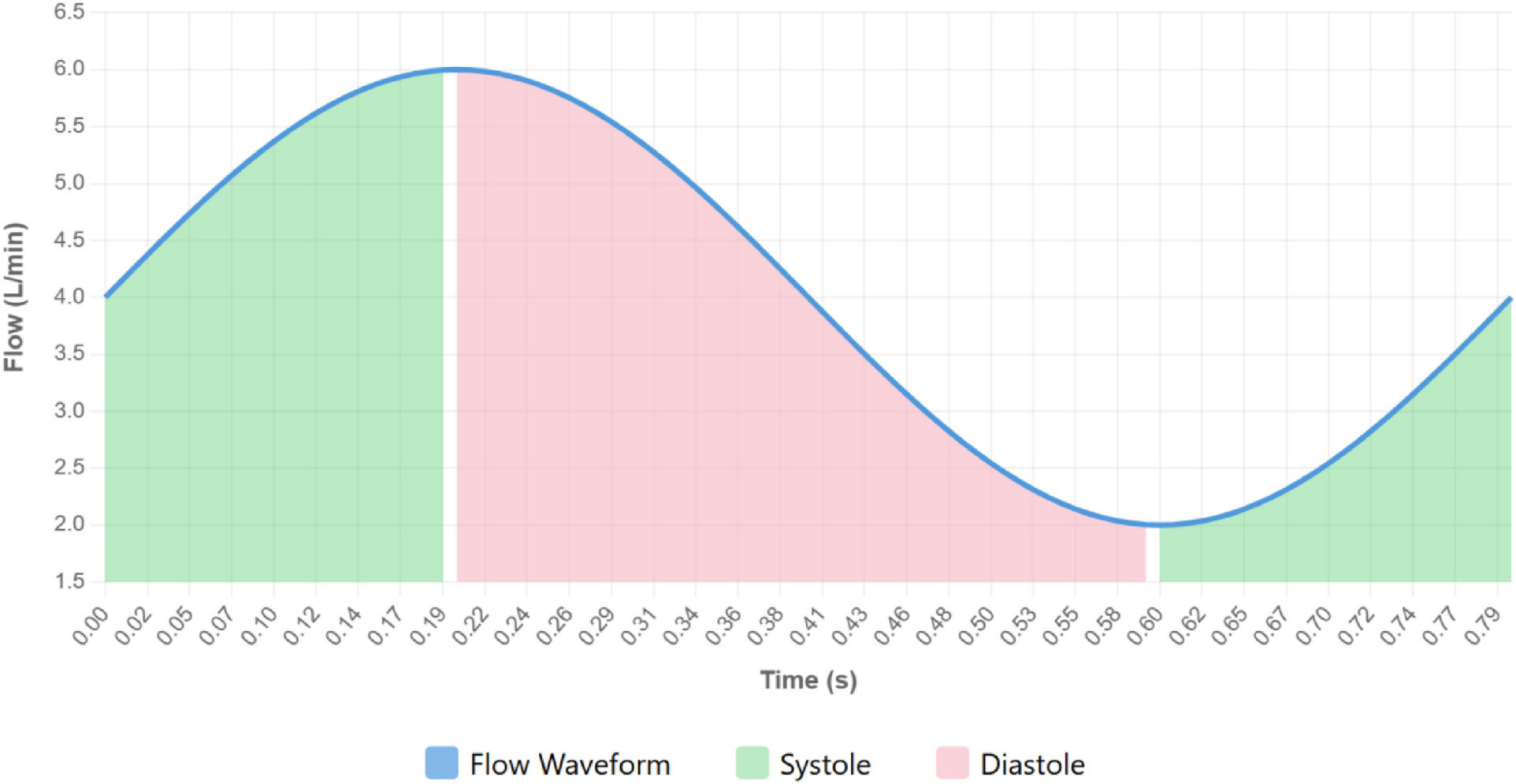
Schematic diagram of the HVAD flow waveform partitioning method [redrawn based on the concepts from Ref. (8)]. *X*-axis: Time (s); *Y*-axis: Flow (L/min). The onset of ventricular systole corresponds to the trough of the flow waveform, and the onset of ventricular diastole corresponds to the peak of the flow waveform.

Owing to the considerable similarity between HeartCon and HVAD in flow computation mechanisms and waveform output characteristics, prior investigations on HVAD offer meaningful guidance for interpreting HeartCon flow waveforms in a physiological context. Research on the HVAD flow waveform has indicated a clear correspondence between the flow waveform and the cardiac cycle: the onset of ventricular systole corresponds to the trough of the flow waveform, and the onset of ventricular diastole corresponds to the peak of the flow waveform, as illustrated in Figure 1 (5). The figure is reproduced from Ref. (8) with permission from the publisher. However, when applying the HVAD partitioning method to the HeartCon flow waveform, we observed a significantly longer systolic duration compared to the diastolic duration, which is inconsistent with normal physiological expectations. To address this issue, this research systematically designed and conducted animal experiments aimed at establishing a more accurate correspondence between the HeartCon pump's flow waveform and the cardiac cycle, ultimately proposing a novel ECG-guided flow waveform cardiac cycle partitioning method. While prior HVAD-based methods relied on identifying the flow trough and peak as systole and diastole onsets, respectively, this approach resulted in a physiologically implausible systolic dominance when applied to the HeartCon waveform. Our method leverages direct ECG synchronization, using the R-wave peak and T-wave end as temporal anchors for systole and diastole, thereby correcting the misalignment observed with HVAD-based partitioning. This addresses a critical scientific gap: the lack of a validated, device-specific waveform partitioning method for the HeartCon pump, which is essential for accurate hemodynamic assessment and clinical monitoring in patients supported by this next-generation LVAD.

Based on the proposed HeartCon flow waveform partitioning method, this research further investigated the effects of different physiological conditions (such as heart rate, blood pressure, rotational speed) on the flow waveform and cardiac cycle, providing a theoretical foundation for future in-depth physiological status assessment and pump function optimization based on the HeartCon flow waveform. The research results not only offer a new perspective for the clinical application of LVADs but also lay the groundwork for developing more intelligent LVAD management strategies.

## 2 Experimental methods

### 2.1 Experimental animals

This investigation selected male adult Small-Tailed Han sheep due to their physiological characteristics and cardiovascular system exhibiting certain similarities to humans, making them a well-established model for cardiovascular device evaluation (9). A total of six male adult Small-Tailed Han sheep (weight: 45–55 kg, age: 2–3 years) were included in this study. All animals completed the experimental protocol without early termination. Inter-animal variability was assessed by calculating the coefficient of variation (CV) for key hemodynamic parameters (e.g., EF, diastolic proportion) across animals under baseline conditions. The CV ranged from 8% to 15%, indicating acceptable inter-subject consistency within this acute model. All experimental animals underwent comprehensive pre-operative physical examinations, including echocardiography and blood parameter analysis (e.g., complete blood count, biochemistry), to confirm the absence of structural cardiac anomalies and systemic diseases. The experimental animal protocol used in this study was approved by the Animal Ethics Committee of the Binhai New Area Medical Experimental Animal Center and strictly adhered to the animal protection regulations of the Binhai New Area Medical Experimental Animal Center.

### 2.2 Experimental reagents and instruments

The essential reagents for this investigation included: succinylcholine (Shanghai Xudong Haipu Pharmaceutical Co., Ltd., anesthetic), sevoflurane (Shanghai Hengrui Pharmaceutical Co., Ltd., inhalational anesthetic), lidocaine hydrochloride injection (Tianjin Jinyao Pharmaceutical Co., Ltd., local anesthetic/antiarrhythmic), dopamine hydrochloride injection (Shanghai Hefeng Pharmaceutical Co., Ltd., vasoactive drug), epinephrine (Tianjin Jinyao Pharmaceutical Co., Ltd., vasoactive drug), norepinephrine injection (Shanghai Hefeng Pharmaceutical Co., Ltd., vasoactive drug), amiodarone hydrochloride injection (Zhejiang Chuangxin Biological Co., Ltd., antiarrhythmic), 10% glucose injection (Otsuka Pharmaceutical Co., Ltd., China, fluid replacement). The vasoactive drugs (dopamine, epinephrine, norepinephrine) were used for blood pressure regulation.

The main instruments included: the HeartCon LVAD system (Rocor Medical, China), MP150 multi-channel physiological recording system (BIOPAC Systems Inc., U.S.), flow sensor (Transonic Systems Inc., U.S.), cardiac pacemaker (Medtronic, Inc., U.S.), color Doppler ultrasound diagnostic system (GE Vingmed Ultrasound AS), and the implantable pressure probes (for LVP, AOP, and LAP measurement).

### 2.3 LVAD and related sensor implantation procedure

The experiment was conducted in the operating room of the Binhai New Area Medical Experimental Animal Center. After successful anesthesia, thoracotomy was performed on the experimental sheep to expose the heart and aorta. A coring needle was used to create an opening at the apex of the left ventricle, and a ventricular sewing ring was sutured in place. After inserting the HeartCon pump, a vascular graft was anastomosed to the distal ascending aorta. To accurately monitor hemodynamic parameters, an aortic pressure (AOP) probe was placed in the aorta, 1–2 cm distal to the graft anastomosis; a flow sensor was placed around the graft near the anastomosis; a left ventricular pressure (LVP) probe was inserted into the left ventricle via the apex; and a left atrial pressure (LAP) probe was placed in the left atrium via puncture of the right atrium. All probes were connected to the MP150 physiological recording system.

### 2.4 Proposal and validation of the HeartCon partitioning method

#### 2.4.1 Heartcon partitioning method

**Figure 2:**
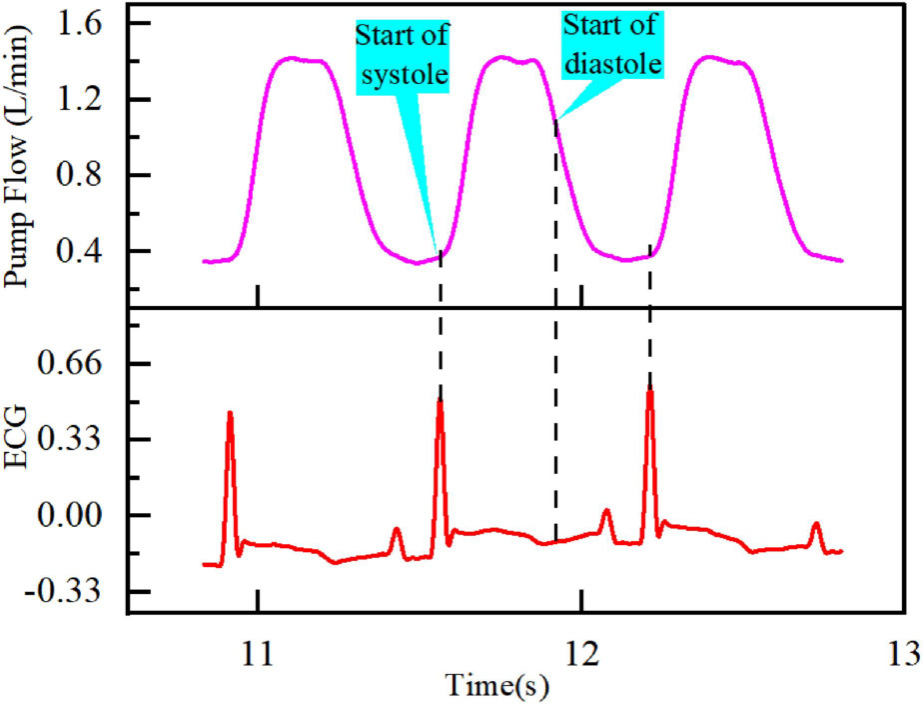
Heartcon partitioning method.

Based on the physiological correlation between the electrocardiogram (ECG) and the flow waveform, this research proposes a method for segmenting the HeartCon flow waveform. Specifically, the peak of the R wave in the ECG is identified as the onset of the systolic phase, corresponding to the initial point of the rapid upstroke in the flow waveform. The end of the T wave is used as the marker for the onset of the diastolic phase, which corresponds to a specific point during the decline phase of the flow waveform (10), as illustrated in Figure 2.

#### 2.4.2 Validation of the HeartCon partitioning method

Prior investigations have indicated that the integral of the HVAD flow waveform effectively reflects pump flow (11). The HVAD calculates flow by monitoring pump current, rotational speed, and patient hematocrit, and displays a real-time flow waveform on the monitor. The principle of its flow calculation is depicted in (Equation 1) (12).

Q=I×RPM×Hct (1)

where, *Q*: Flow; *I*: electric current; *RPM*: speed; *Hct*: hematocrit (8).

**Figure 3:**
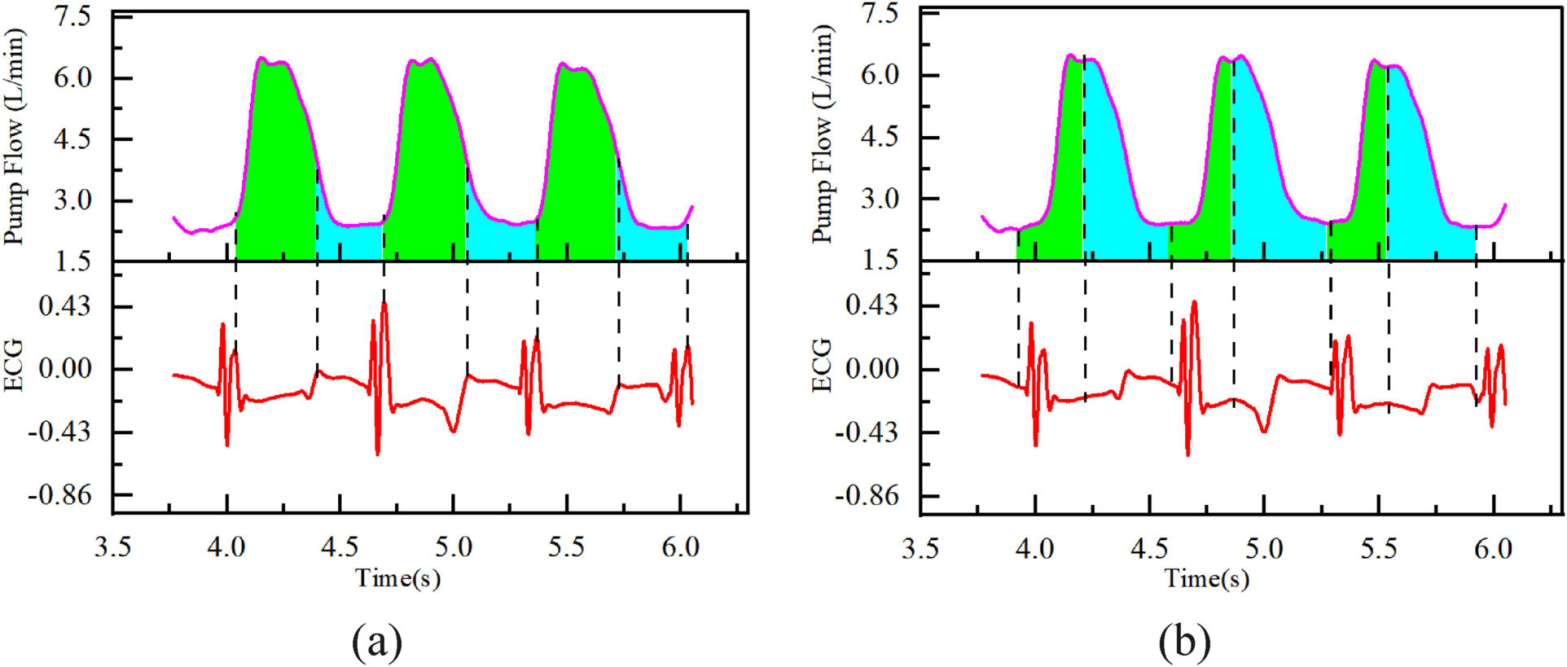
Integral method **(a)** heartCon partitioning method; **(b)** HVAD partitioning method; green area: systole (*Q_S_*); blue area: diastole (*Q_D_*).

Given the similarities between HeartCon and HVAD in flow calculation principles and flow waveform output methods, this research used the HeartCon flow waveform and applied both the proposed HeartCon partitioning method and the previously reported HVAD partitioning method to partition flow waveforms at different rotational speeds (2,300–2,700 RPM). Origin software was used to calculate the systolic flow integral (*Q_S_*) and diastolic flow integral (*Q_D_*), as depicted in Figure 3.

Subsequently, the ejection fraction (EF) was calculated for both partitioning methods using the formula proposed by Kiehl et al. (11) (Equation 2), and the calculated EF values were compared with the EF values measured by color Doppler ultrasound during the experiment. All ultrasound examinations were performed by a single, experienced cardiac sonographer who was blinded to the partitioning method-derived EF values. The EF was measured using the biplane Simpson's method of discs, in accordance with current guidelines (13).

$$EF=Q_{S}/(Q_{S}+Q_{D})\times 100\text{\%}$$ (2)

where, *Q_S_*: systolic flow integral; *Q_D_*: diastolic flow integral (7).

Due to the focus of this manuscript on the methodological validation and waveform analysis, and considering the space limitations, images of the HeartCon and HVAD devices and their display interfaces are not included here. However, the functional output (i.e., the flow waveform) of these devices is the central subject of our analysis and is depicted in the presented figures. Future publications may include device images for completeness.

### 2.5 Effects of different conditions on flow waveform and cardiac cycle

While maintaining stable basic physiological parameters of the experimental animals, the pump rotational speed was adjusted to 2,400, 2,500, 2,600, 2,700, and 2,800 RPM, covering the typical operating range for the HeartCon pump in preclinical settings based on preliminary tests and manufacturer recommendations, and ensuring a comprehensive assessment of speed-dependent effects on hemodynamics to investigate the effects of different rotational speeds on the flow waveform and cardiac cycle. The cardiac pacemaker frequency was adjusted to stabilize the experimental animals' heart rates at 62, 76, 90, 105, and 119 BPM, representing a clinically relevant spectrum from bradycardia to tachycardia often encountered in heart failure patients managed with LVADs (6, 8) to investigate the effects of different heart rates on the flow waveform and cardiac cycle. By adjusting the infusion rate of vasoactive drugs, the experimental animals' mean arterial pressure (MAP) was regulated to 67, 75, and 85 mmHg to investigate the effects of different blood pressures on the flow waveform and cardiac cycle. Under each set condition, physiological parameters such as pump flow, LAP, LVP, AOP, and ECG were simultaneously collected, and the flow waveform was partitioned using the proposed HeartCon partitioning method to analyze the correspondence between the cardiac cycle and the flow waveform, and to calculate the time proportion of systole and diastole in the entire cardiac cycle.

### 2.6 Data acquisition

Hemodynamic information such as pump flow (obtained through a flow sensor), LAP, LVP, and AOP, as well as ECG signals, pump current, and voltage, were simultaneously collected using the MP150 multi-channel physiological recording system. The sampling frequency for all signals was set to 1,000 Hz.

### 2.7 Data processing and analysis

The relative error (δ) was used to evaluate the difference between the EF values obtained by the HeartCon and HVAD partitioning methods and the ultrasound EF values (EFUS), calculated using (Equation 3). To provide a more comprehensive statistical validation, we also performed Bland-Altman analysis to assess agreement between methods, calculated the root mean squared error (RMSE) and mean absolute error (MAE), and computed Pearson correlation coefficients. Paired *t*-tests were used to compare EF values derived from the HeartCon method versus ultrasound, with *p* < 0.05 considered statistically significant. These analyses were conducted using SPSS version 26.0 (IBM Corp., Armonk, NY, USA).

$$\delta =(\bar{EF}-EF_{\mathrm{US}})EF_{\mathrm{US}}\times 100\text{\%}$$ (3)

where, $\bar{EF}$ is the calculated average *EF* value using a specific partitioning method.

## 3 Experimental results

### 3.1 Validation of the HeartCon partitioning method

**Figure 4:**
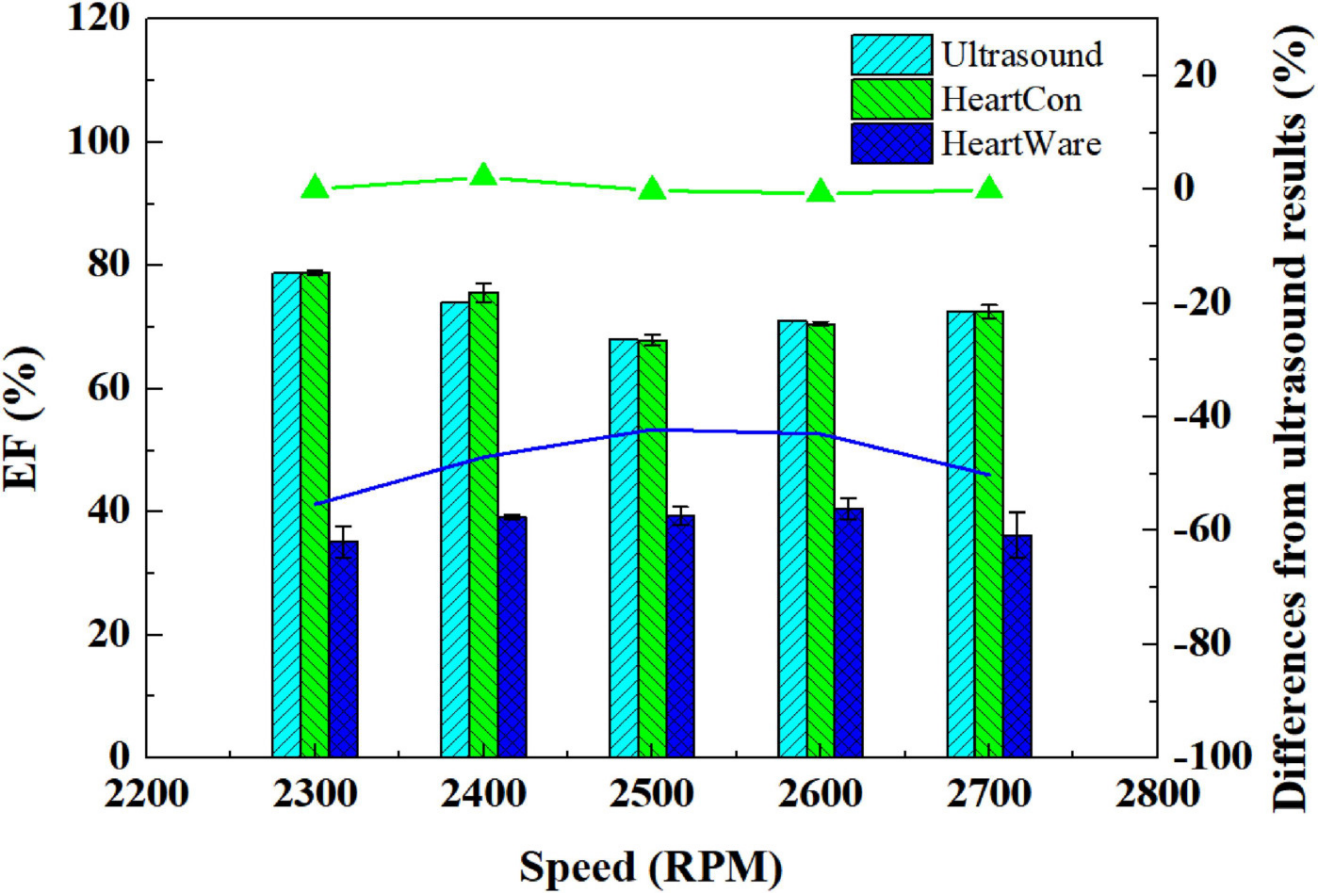
Comparison of ejection fraction (EF) values derived from the heartCon partitioning method, HVAD method, and ultrasound (reference) across different pump speeds (2,300–2,700 RPM). Data are presented as mean ± SD (*n* = 6 animals). The HeartCon method shows close agreement with ultrasound, whereas the HVAD method consistently underestimates EF. Units: EF (%).

Comparative analysis revealed that under different rotational speeds, the absolute relative errors of EF values obtained by the HeartCon partitioning method were significantly below 5%, while the absolute relative errors in the HVAD group exceeded 40%. Furthermore, the errors in the HeartCon group showed alternating positive and negative values, suggesting predominantly random errors, whereas the HVAD group exhibited consistent negative errors, indicating a need for systematic correction. As presented in Figure 4, the EF values measured by the HeartCon partitioning method demonstrated good agreement and high repeatability with the ultrasound results. Statistical analysis demonstrated excellent agreement between the HeartCon-derived EF and ultrasound EF, with a mean bias of 0.8% (95% limits of agreement: −3.2% to 4.8%) in Bland-Altman analysis, RMSE of 2.1%, MAE of 1.7%, and a correlation coefficient of *r* = 0.96 (*p* < 0.001). In contrast, the HVAD method showed poor agreement (mean bias: −18.5%, 95% LoA: −32.1% to −4.9%, RMSE: 20.3%, MAE: 18.7%, *r* = 0.42, *p* = 0.12). These results confirm the superior accuracy and reliability of the proposed HeartCon partitioning method.

### 3.2 Effects of different conditions on flow waveform and cardiac cycle

#### 3.2.1 Effects of different heart rates on flow waveform and cardiac cycle

**Figure 5:**
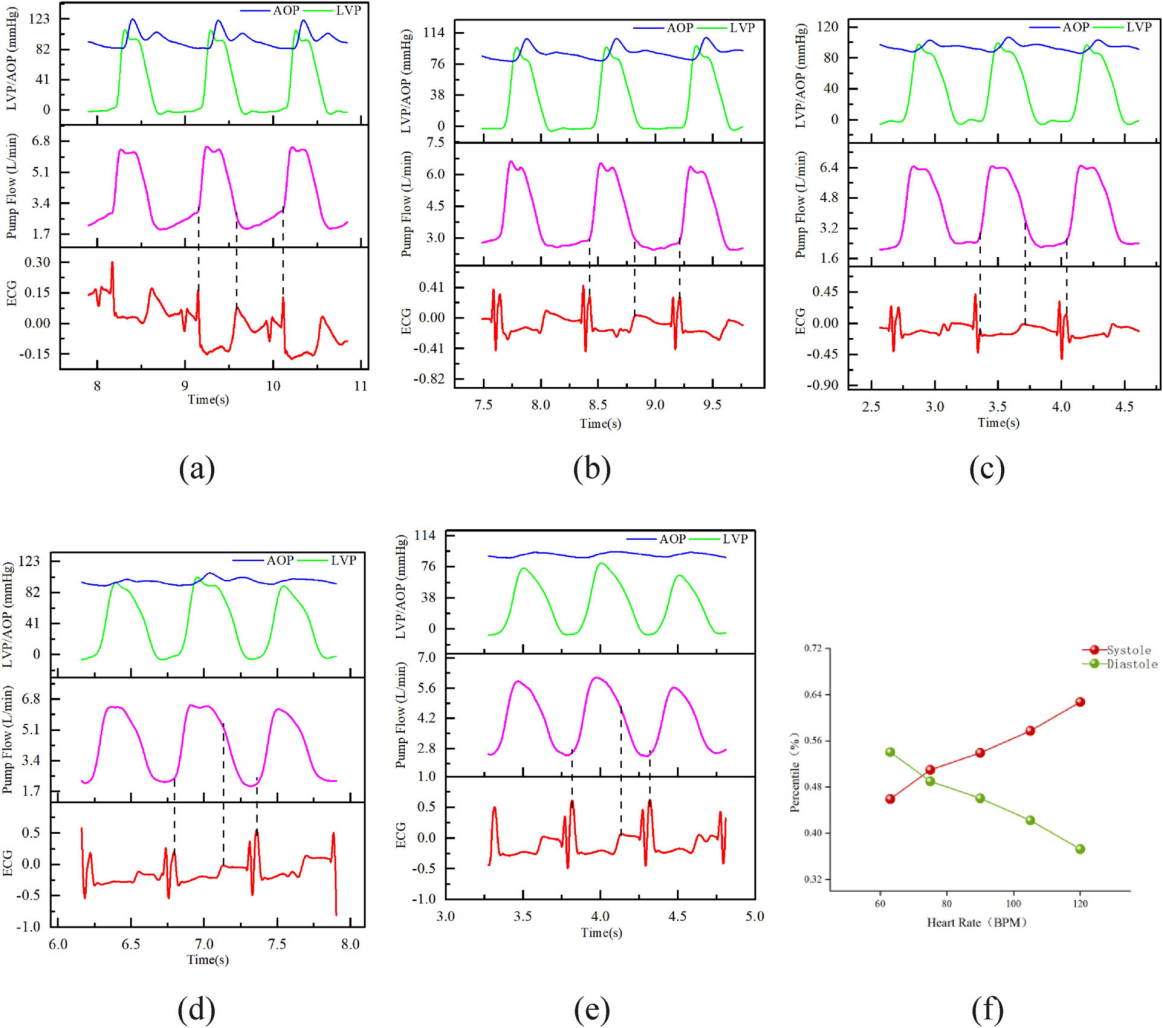
Result chart of the influence of different heart rates on flow waveform and cardiac cycle. Fixed conditions: speed 2,600 RPM, MAP ≈ 95 mmHg; **(A)** heart rate 62 BPM; **(B)** heart rate 76 BPM; **(C)** heart rate 90 BPM; **(D)** heart rate 105 BPM; **(E)** heart rate 119 BPM; **(F)** The proportion of diastolic phase in the entire cardiac cycle at different heart rates.

The flow waveform was partitioned according to the HeartCon partitioning method under the fixed conditions of rotational speed (2,600 RPM) and MAP (approximately 95 mmHg). As shown in Figures 5A–E, the results demonstrated that with increasing heart rate, the onset of systole in the flow waveform remained consistently at the point of rapid flow increase, while the onset of diastole gradually approached the peak of the flow waveform. Simultaneously, a gradual decrease in the aortic valve opening amplitude (judged by the difference between LVP and AOP) was observed.

Based on Figures 5A–E, the proportions of systole and diastole in the entire cardiac cycle were calculated, as shown in Figure 5F. The diastolic phase proportion showed a significant downward trend with increasing heart rate and was less than the 58% diastolic proportion in a normal heart (13) at all tested heart rates.

#### 3.2.2 Effects of different blood pressures on flow waveform and cardiac cycle

**Figure 6:**
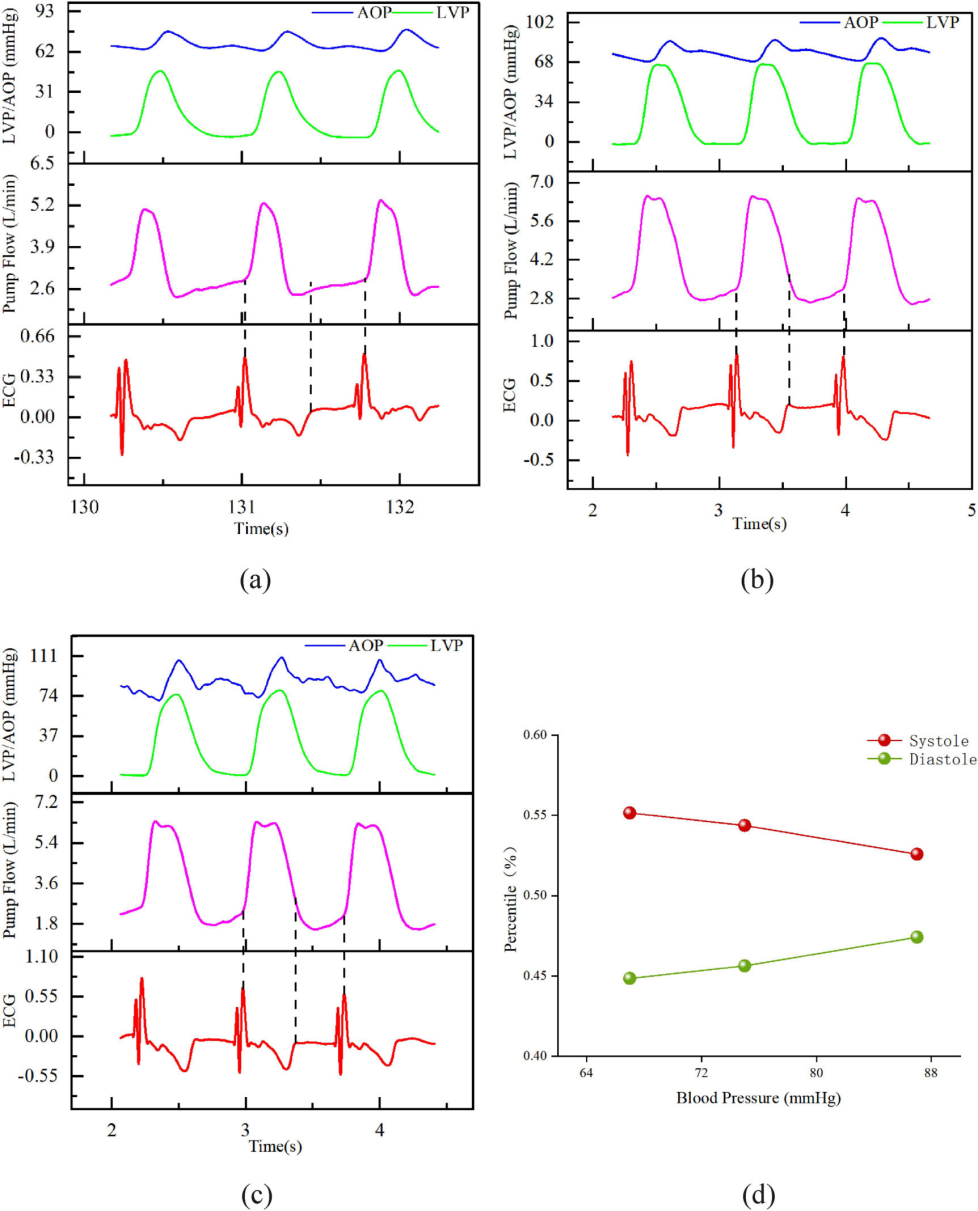
Result chart of the influence of different blood pressures on flow waveform and cardiac cycle. Fixed conditions: speed 2,400 RPM, heart rate 70–80 bpm; **(A)** MAP 67 mmHg; **(B)** MAP 75 mmHg; **(C)** MAP 85 mmHg. **(D)** The proportion of diastolic phase in the entire cardiac cycle under different blood pressures.

Under fixed rotational speed (2,400 RPM) and heart rate (70–80 BPM), the MAP was adjusted to 67, 75, and 85 mmHg. The schematic diagrams of flow waveform partitioning are shown in Figures 6A–C. As the MAP increased, the starting point of diastole in the flow waveform gradually approached the peak. Figure 6D shows that the diastolic phase proportion tended to increase with increasing blood pressure, but all values were significantly less than 58%.

#### 3.2.3 Effects of different rotational speeds on flow waveform and cardiac cycle

**Figure 7:**
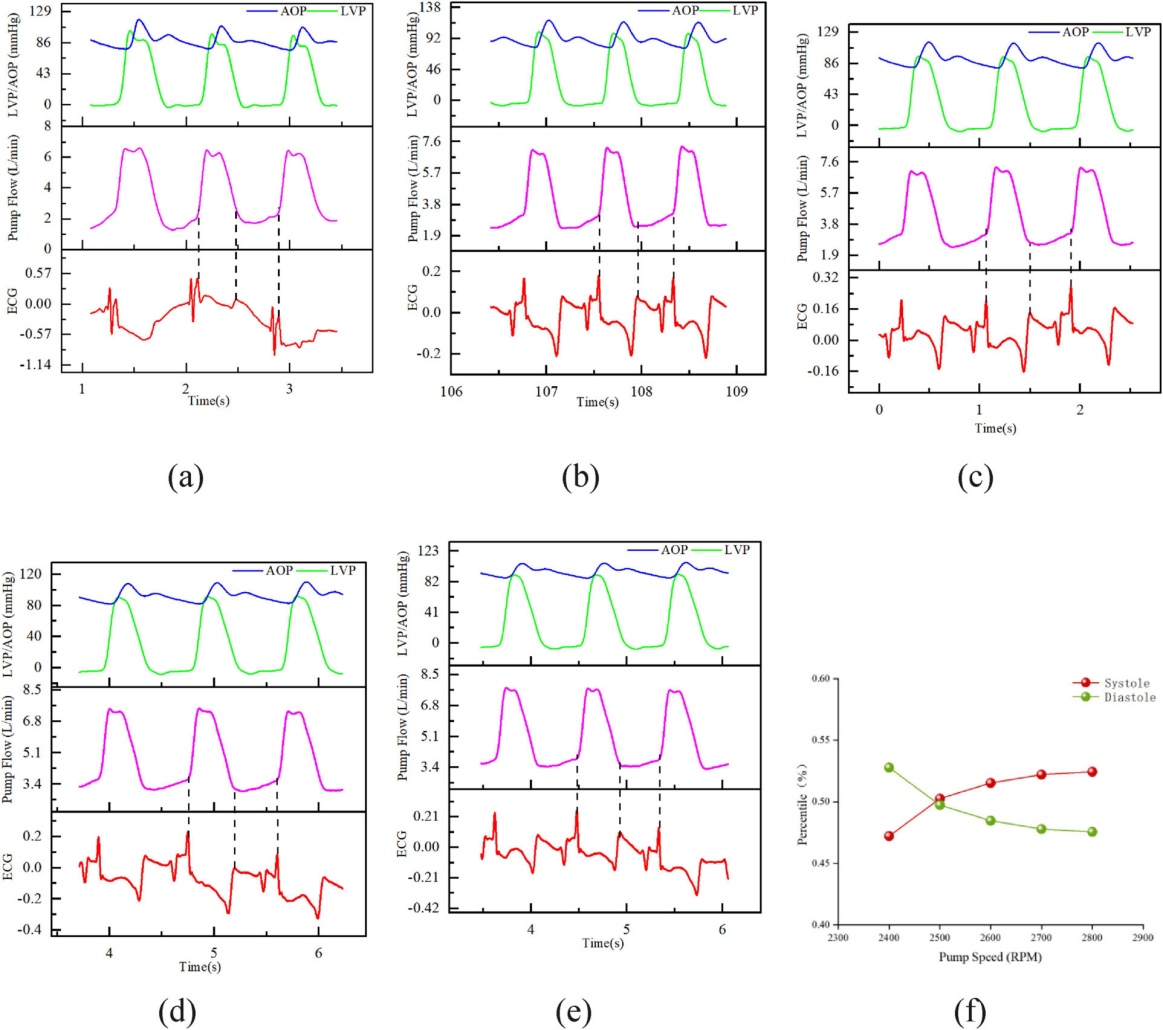
Result chart of the influence of different speeds on flow waveform and cardiac cycle. Fixed conditions: heart rate 70–80 bpm; MAP ≈ 92 mmHg; **(A)** speed 2,400 RPM; **(B)** speed 2,500 RPM; **(C)** speed 2,600 RPM; **(D)** speed 2,700 RPM; **(E)** speed 2,800 RPM; **(F)** The proportion of diastolic phase in the entire cardiac cycle at different speeds.

Under fixed heart rate (70–80 BPM) and MAP (approximately 92 mmHg), the pump rotational speed was adjusted to 2,400, 2,500, 2,600, 2,700, and 2,800 RPM. The schematic diagrams of flow waveform partitioning are shown in Figures 7A–E. As the rotational speed increased, the starting point of diastole in the flow waveform gradually approached the trough. Figure 7F shows that the proportion of diastole in the entire cardiac cycle gradually decreased with increasing rotational speed, and all values were significantly less than 58%.

#### 3.2.4 Heartcon cardiac Wiggers diagram

**Figure 8:**
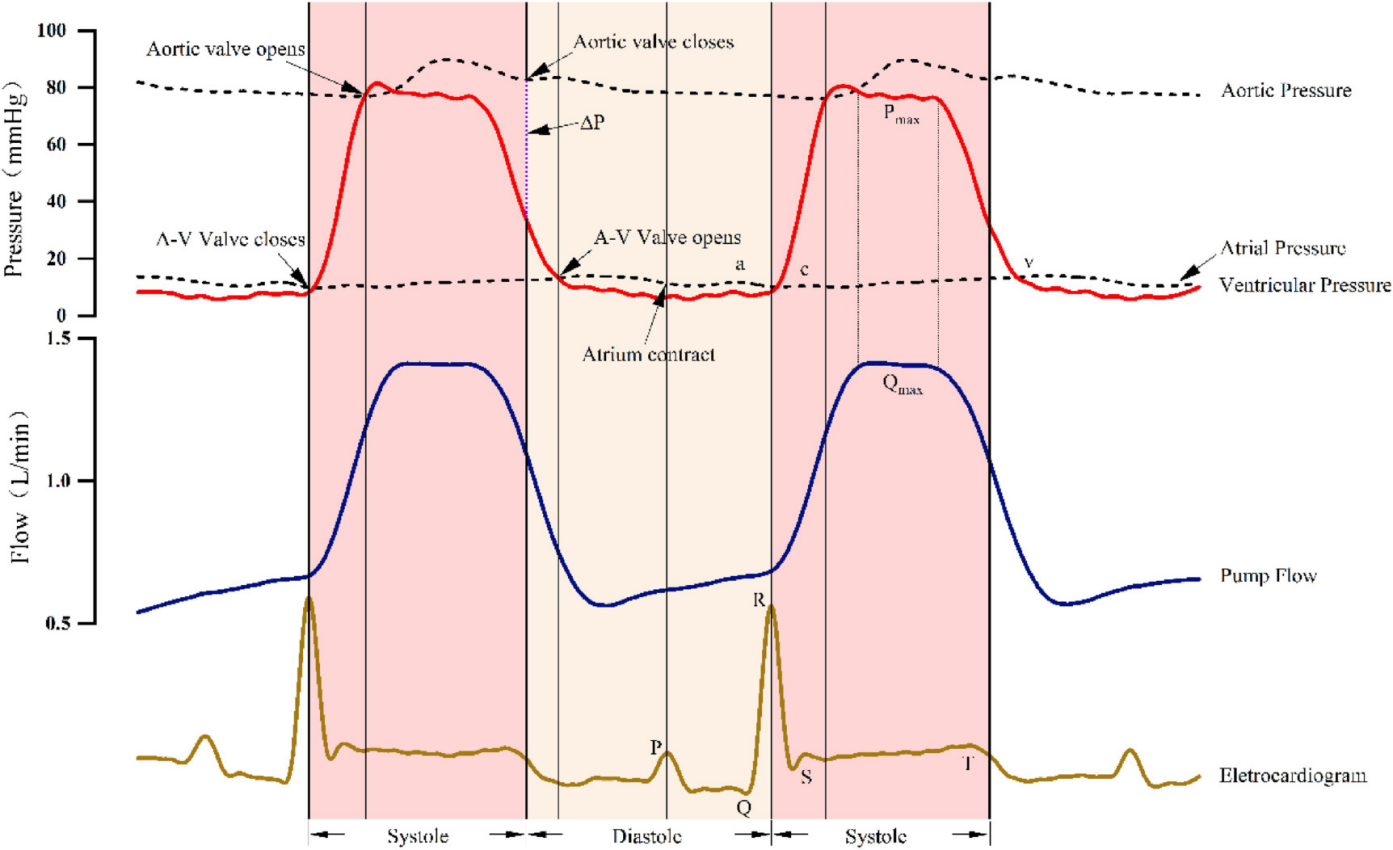
HeartCon-specific wiggers diagram under representative conditions (speed 2,400 RPM, heart rate 90 BPM, MAP 77 mmHg). Curves include ECG (top), pump flow (L/min), left ventricular pressure (LVP, mmHg), aortic pressure (AOP, mmHg), and left atrial pressure (LAP, mmHg). Systole (yellow shading) and diastole (blue shading) are demarcated using the proposed ECG-guided partitioning method. Note the attenuated atrial waves (*a*, *c*, *v*) and the flat-topped flow peak (QMAX).

**Figure 9:**
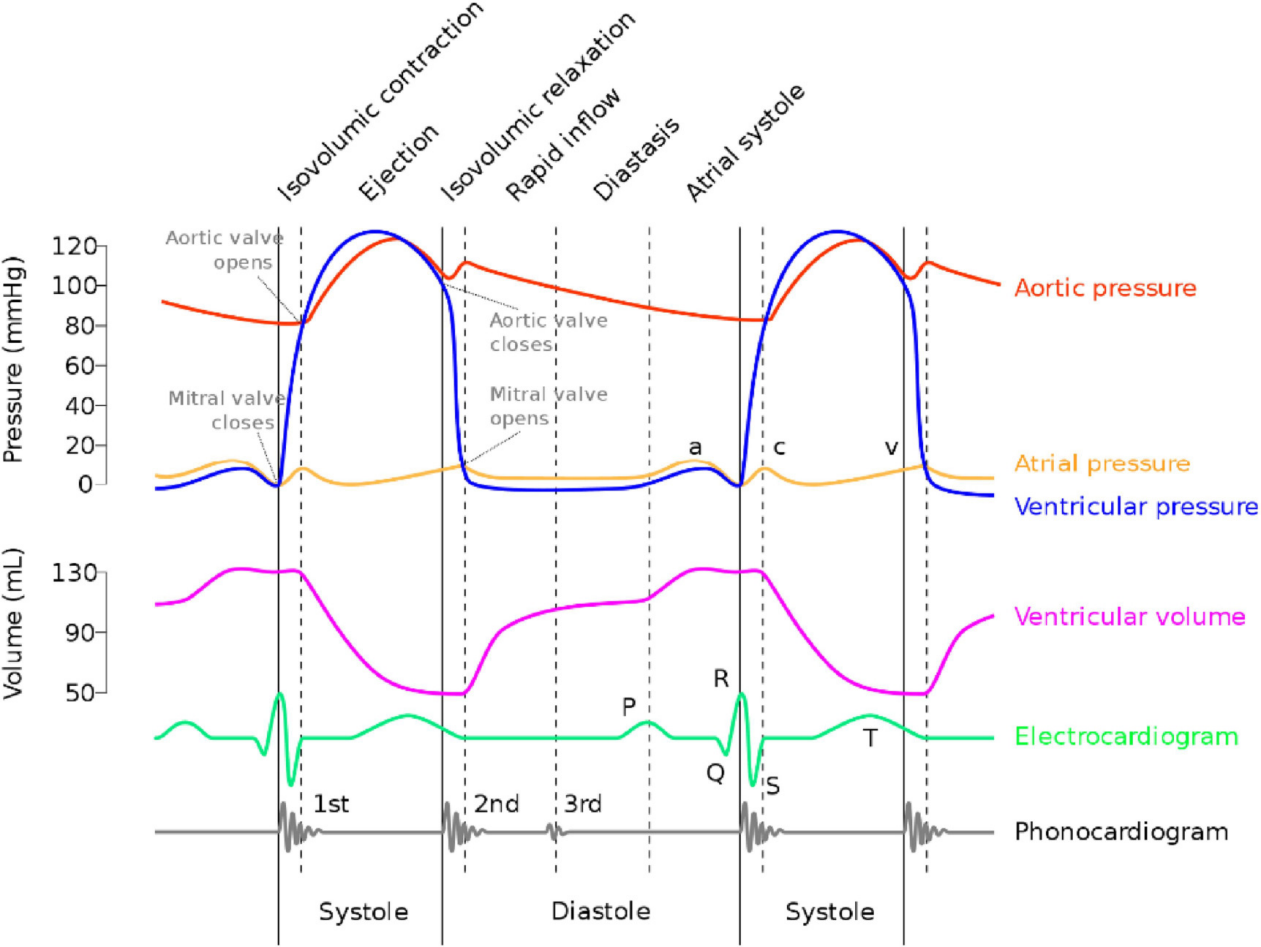
Schematic of a classic normal wiggers diagram. This is an original schematic created by the authors based on standard physiological concepts and descriptions found in authoritative medical physiology textbooks (14), included for illustrative and comparative purposes.

A representative operating condition (e.g., rotational speed 2,400 RPM, heart rate 90 BPM, MAP 77 mmHg) was selected, and a HeartCon cardiac Wiggers diagram after implantation was constructed based on the HeartCon partitioning method, as shown in Figure 8. To provide a physiological reference, a schematic of a classic normal Wiggers diagram is included as Figure 9. Figure 9 is an original schematic created by the authors based on standard physiological descriptions found in authoritative texts (e.g., Guyton and Hall Textbook of Medical Physiology) (14) to ensure clarity and avoid copyright issues. It includes the typical curves: electrocardiogram (ECG), aortic pressure (AOP), left ventricular pressure (LVP), left atrial pressure (LAP), ventricular volume, and phonocardiogram (heart sounds).

In contrast, our HeartCon Wiggers diagram (Figure 8) is adapted to the context of LVAD support: it omits the ventricular volume curve and phonocardiogram due to the focus on pump interaction and includes the pump flow curve to more clearly illustrate the temporal correspondence between pump flow, ECG, and pressures (LVP, AOP, LAP). Key physiological and supraphysiologic changes induced by the HeartCon LVAD are evident in the Wiggers diagram.

Key physiological and supraphysiologic changes induced by the HeartCon LVAD are evident in the Wiggers diagram. In the LVP curve, the starting point of the sharp rise closely corresponds to the starting point of systole in the flow waveform (the peak of the R wave in the ECG). In the AOP curve, the peak of the dicrotic notch, which marks the closure of the aortic valve, coincides with the specific point after the rapid decline in the flow waveform and the end of the T wave in the ECG. The pressure curves directly illustrate the impact of LVAD implantation: due to the continuous unloading of the left ventricle by the pump, the pressure difference (ΔP) between LVP and AOP during aortic valve opening is significantly increased, representing a supraphysiologic alteration in ventricular afterload. Furthermore, the characteristic atrial pressure waves are attenuated: the a wave of the LAP curve is diminutive, the c wave is nearly absent, and the v wave is delayed and blunted. Notably, the systolic and diastolic time intervals determined by the HeartCon partitioning method are highlighted with shading or vertical lines in Figure 8 for a more intuitive understanding of the partitioning method's representation on the Wiggers diagram.

Additionally, The pump flow curve exhibits a flat peak (*Q* ∼ MAX∼) characteristic rather than a typical pulsatile sinusoidal waveform, which could suggest that the pump's output approaches its maximum capacity under the current operating parameters, although this is not directly proven here. Further analysis shows that the peak of AOP occurs in the middle of the flow waveform peak, indicating that the pump starts to output its maximum flow before the peak of ventricular systolic energy is reached; during the sustained rise of AOP, the pump flow shows no significant change, consistent with the hypothesis of pump output saturation, yet alternative explanations such as altered ventricular-pump coupling cannot be excluded.

The pump flow curve exhibits a flat peak (*Q*_MAX_) characteristic rather than a typical pulsatile sinusoidal waveform, suggesting that the pump's output may have approached its maximum capacity under the current operating parameters. Further analysis shows that the peak of AOP occurs in the middle of the flow waveform peak, indicating that the pump starts to output its maximum flow before the peak of ventricular systolic energy is reached; during the sustained rise of AOP, the pump flow shows no significant change, further confirming that the pump is operating at its maximum output under the current parameters.

## 4 Discussion

### 4.1 Effects of different conditions on flow waveform and cardiac cycle

Our findings align with and extend prior reports on continuous-flow LVAD hemodynamics. Similar to HeartMate II/III studies, we observed attenuation of atrial pressure waves (*a*, *c*, *v* waves) in the LAP curve, consistent with ventricular unloading and reduced atrial stretch. The flat-topped flow waveform (QMAX) resembles the “flow plateau” reported in HVAD studies during high-speed operation or hypovolemia. However, unlike HVAD suction events characterized by sharp flow dips and elevated pump power, we did not observe overt suction in this acute setting, possibly due to careful speed titration. The shortened diastolic proportion is a novel observation specific to HeartCon, suggesting device-specific hemodynamic interactions that warrant comparison with HeartMate II/III data in future studies. Overall, our results reinforce that LVAD support fundamentally alters cardiac timing and pressure morphology, but device-specific features must be recognized for accurate clinical interpretation.

This investigation methodically assessed the effects of changes in heart rate, blood pressure, and LVAD rotational speed on the HeartCon flow waveform and cardiac cycle by controlling these parameters in experimental animal. The results show that an increased heart rate significantly shortens the proportion of the cardiac cycle occupied by diastole, potentially leading to insufficient ventricular filling, which in turn may affect pump flow and reduce the amplitude of aortic valve opening. Under hypotensive conditions, the pump's afterload is reduced, resulting in increased pump flow. However, a decrease in the diastolic phase proportion was also observed, possibly due to enhanced pump aspiration force affecting ventricular diastolic function. When the LVAD rotational speed is increased, both the pump's output flow and aspiration force increase, further shortening the diastolic phase proportion (11), suggesting that excessively high pump speeds may excessively unload the left ventricle and interfere with its normal filling. Notably, the diastolic phase proportion was significantly lower than the 58% in a normal heart (15) under all tested conditions, which may be related to the hemodynamic changes after LVAD implantation and the continuous flow support provided by the pump. The observed shortening of diastole raises the hypothesis that it may adversely affect myocardial perfusion, since coronary perfusion occurs predominantly during diastole, driven by the aortic diastolic pressure that generates the critical perfusion gradient across the coronary vascular bed (16–20). While physiological and supraphysiological augmentation of diastolic pressure and duration are recognized mechanisms for enhancing coronary flow (20, 21–23), whether the abbreviated diastole under HeartCon support directly compromises coronary perfusion remains speculative and requires direct measurement of coronary flow in future studies. Previous studies have shown that in normal physiological conditions, the duration of ventricular systole is approximately 330 ± 42 ms, and diastole is approximately 469 ± 76 ms. Diastole is significantly longer than systole, accounting for about 58% of the cardiac cycle, which is considered crucial for maintaining sufficient cardiac preload and ensuring effective coronary artery perfusion (15). For HF patients, appropriately prolonging diastole has been associated with improved left ventricular reverse remodeling and patient prognosis (24). Our data suggest that the optimal diastolic phase proportion for patients with implanted LVADs may differ from the normal reference value of 58%; however, this remains a hypothesis that requires validation in future clinical studies. The associations between prolonged tachycardia, persistent hypotension, or indiscriminate increases in LVAD rotational speed and impaired postoperative cardiac function recovery in HF patients (6) are plausible but speculative based on our acute experimental data, warranting confirmation in chronic clinical settings.

### 4.2 Potential reasons for T-wave inversion

The phenomenon of T-wave inversion observed in the ECG during the experiment is consistent with findings in previous studies using other animal models (25). T-wave inversion observed in our experiments likely resulted from multiple factors specific to our model: (1) anesthetic agents such as sevoflurane and opioids are known to affect ventricular repolarization; (2) surgical trauma from apical coring and suturing may cause local myocardial injury or edema, altering depolarization/repolarization vectors; (3) hemodynamic shifts due to LVAD unloading could change wall stress and consequently affect repolarization; (4) possible electrolyte imbalances from fluid shifts or drug infusions (e.g., dopamine) may contribute. While we did not perform detailed electrophysiological mapping, the consistent presence of T-wave inversion across animals suggests a reproducible effect of the acute implant model rather than random variation. Future studies incorporating continuous electrolyte monitoring and high-resolution mapping could elucidate the precise mechanisms (26). The specific mechanisms of T-wave inversion were not deeply investigated in this research and require further electrophysiological studies to elucidate.

### 4.3 Consideration of electromechanical delay

The electromechanical delay (EMD), typically 25–40 ms in humans and similar large animals, represents the interval between electrical depolarization (R-wave) and mechanical contraction onset (pressure rise). In our study, we observed an EMD of 30 ms between the ECG R-wave and the rapid flow upstroke, consistent with published physiology. This delay has important implications for clinical waveform interpretation: it means that the flow waveform lags behind electrical events. Therefore, using ECG landmarks (R-wave peak, T-wave end) as temporal anchors for partitioning inherently accounts for this physiological delay, ensuring that mechanical phases are correctly aligned. Ignoring EMD could lead to misidentification of systolic onset, especially in patients with conduction abnormalities or on inotropic support. Future implementations of automated waveform analysis algorithms should incorporate EMD correction for enhanced accuracy (27). This physiological phenomenon was considered when analyzing the relationship between the ECG and the flow waveform. However, since a time interval of 30 ms is difficult to visually distinguish directly on the HeartCon monitor interface, for the convenience of clinical application and waveform interpretation, this study simplifies the point of rapid flow increase as the onset of systole.

### 4.4 Clinical significance and limitations

The primary significance of constructing the HeartCon cardiac Wiggers diagram lies in establishing a preliminary temporal relationship between the flow waveform and key hemodynamic parameters, clearly demonstrating the significant differences between cardiac function after LVAD implantation and that of a normal heart. This lays the foundation for subsequent research on the characteristic patterns of flow waveforms in LVAD patients under different physiological or pathological conditions. These characteristics may manifest as specific waveform morphologies, changes in key time points, or abnormalities in certain characteristic parameters.

Notably, the presence of a flat-topped QMAX characteristic in the flow waveform may indicate that the pump is operating near its maximum output under the tested conditions; however, this interpretation is preliminary and requires further validation under varied loading states. The rotational speeds used (2,400–2,800 RPM) in this acute animal model were selected based on preliminary tests and manufacturer recommendations to cover the typical operating range for the HeartCon pump in preclinical settings. While the ovine cardiovascular system shares similarities with humans (6), the acute nature of this study and potential species-specific differences mean that the absolute speed values and their precise hemodynamic effects may not directly and fully replicate the chronic adaptive physiology of the human heart with an implanted LVAD. Future chronic studies in appropriate models are needed to better simulate long-term human physiology. Further analysis indicates that the peak of AOP occurs in the middle of the flow waveform peak, implying that the rapid rise in pump flow precedes the peak of ventricular systolic energy; during the sustained rise of AOP, the pump flow remains relatively stable, further supporting the inference that the pump is operating at maximum output. In situations where myocardial contractility is still adequate but circulatory volume is insufficient, *Q*_MAX_ is expected to terminate at the peak of the AOP waveform, with a narrower width compared to that observed under volume-replete conditions. Future research could focus on investigating the flow waveform characteristics under different combinations of pump operating parameters and various physiological states to establish quantitative assessment criteria for parameters such as *Q*_MAX_ width, providing clinicians with a reference for preliminary judgment of patients' volume status based on the flow waveform. Regarding the potential application of this method to assess ejection fraction in normal hearts without LVADs using pulse pressure information, the current partitioning method is specifically designed for and validated in the context of continuous-flow LVAD support, where the pump flow waveform morphology is fundamentally different from the arterial pressure waveform or native cardiac output patterns. Therefore, direct extrapolation to non-LVAD hearts is not supported by the present data and would require separate methodological development and validation.

However, this research also has certain limitations. First, the observation period in the animal experiments was relatively limited, which may not fully simulate the adaptive changes of the heart after long-term LVAD implantation. Second, the strong aspiration force generated by the pump may have masked some subtle changes in the flow waveform, limiting the observation and analysis of more nuanced physiological events. As shown in Figure 8, compared with the normal cardiac Wiggers diagram (11), the characteristic *a*, *c*, and *v* waves reflecting atrial pressure changes are markedly attenuated in the HeartCon cardiac Wiggers diagram. On the current flow waveform, only the feature point corresponding to mitral valve closure and the associated abrupt rise in flow can be identified, while the opening and closing points of the other valves cannot be clearly annotated. Furthermore, key parameters in heart failure management such as central venous pressure (CVP) and pulmonary capillary wedge pressure (PCWP), which reflect right heart function and left-sided filling pressures respectively, were not extensively analyzed in relation to the flow waveform in this initial study. These limitations suggest that certain abnormal physiological conditions, including those related to venous return and biventricular interactions, may not be directly evident in the current flow waveform, and a comprehensive assessment combining other monitoring methods, such as simultaneous CVP and PCWP measurement, will be necessary in the future.

## 5 Limitations

This study has several limitations. First, it employed an acute ovine model, which does not replicate the chronic adaptive changes (e.g., reverse remodeling, fibrosis) seen in human LVAD recipients. Second, we did not systematically modulate preload (e.g., volume loading) or afterload (e.g., vasoconstrictor titration), which limits the generalizability of the waveform responses across the full physiological spectrum. Third, key filling pressures such as CVP and PCWP were not measured, precluding correlation of flow waveform features with right heart function or left-sided filling status. Fourth, the sample size was modest (*n* = 6), though consistent with acute large-animal device studies. Fifth, the observation period was short, and long-term effects of the partitioning method on clinical outcomes remain unknown. Future studies should address these gaps through chronic models, comprehensive hemodynamic profiling, and clinical validation in patients.

## 6 Conclusion

This investigation successfully proposed a novel ECG-guided flow waveform cardiac cycle partitioning method for the HeartCon pump and validated its rationality and accuracy in assessing ejection fraction through animal experiments, significantly outperforming the previously used HVAD-based partitioning method. The experimental results further revealed that under different physiological conditions, the diastolic phase proportion in animals with implanted HeartCon pumps was significantly lower than normal physiological values and was significantly affected by heart rate, blood pressure, and pump rotational speed, suggesting that pump aspiration force may play an important role in cardiac diastolic function. Given the importance of diastole for cardiac preload and coronary artery perfusion, prolonged tachycardia, hypotension, or blindly increasing pump speed may be detrimental to cardiac function recovery after LVAD implantation in HF patients. By constructing a HeartCon cardiac Wiggers diagram and analyzing its waveform characteristics, the flat-topped Q∼MAX∼ feature in the flow waveform was identified as a potential correlate of pump output dynamics; its relationship with patient volume status remains hypothetical and should be tested in future studies correlating QMAX morphology with direct measures of preload such as PCWP or volume challenge responses. Future research directions could include exploring the optimal diastolic phase proportion range for LVAD patients and investigating whether volume assessment criteria can be established based on Q∼MAX∼ and other flow waveform characteristic parameters, thereby potentially providing clinicians with more reliable bases for inferring patient physiological information, including volume status, based on real-time HeartCon flow waveforms.

## Data Availability

The original contributions presented in the study are included in the article/Supplementary Material, further inquiries can be directed to the corresponding authors.
